# Growth of Monolayer MoS_2_ Flakes via Close Proximity Re-Evaporation

**DOI:** 10.3390/nano14141213

**Published:** 2024-07-17

**Authors:** Blagovest Napoleonov, Dimitrina Petrova, Nikolay Minev, Peter Rafailov, Vladimira Videva, Daniela Karashanova, Bogdan Ranguelov, Stela Atanasova-Vladimirova, Velichka Strijkova, Deyan Dimov, Dimitre Dimitrov, Vera Marinova

**Affiliations:** 1 Institute of Optical Materials and Technologies, Bulgarian Academy of Sciences, 1113 Sofia, Bulgaria; bnapoleonov@iomt.bas.bg (B.N.); dpkerina@gmail.com (D.P.); vvideva@iomt.bas.bg (V.V.); adi@iomt.bas.bg (D.K.); vili@iomt.bas.bg (V.S.); dean@iomt.bas.bg (D.D.); ddimitrov@iomt.bas.bg (D.D.); 2 Faculty of Engineering, South-West University “Neofit Rilski”, 2700 Blagoevgrad, Bulgaria; 3 Institute of Solid State Physics, Bulgarian Academy of Sciences, 1784 Sofia, Bulgaria; rafailov@issp.bas.bg; 4 Faculty of Chemistry and Pharmacy, Sofia University, 1 James Bourchier Blvd., 1164 Sofia, Bulgaria; 5 Institute of Physical Chemistry, Bulgarian Academy of Sciences, 1113 Sofia, Bulgaria; rangelov@ipc.bas.bg (B.R.); statanasova@ipc.bas.bg (S.A.-V.); 6 Department of Physics, University of Chemical Technology and Metallurgy, 8 Kl. Ohridski Blvd., 1756 Sofia, Bulgaria

**Keywords:** synthesis of transition metal dichalcogenides, MoS_2_ nanoflakes, re-evaporation, structural properties, optical properties, Raman analysis

## Abstract

We report a two-step growth process of MoS_2_ nanoflakes using a low-pressure chemical vapor deposition technique. In the first step, a MoS_2_ layer was synthesized on a c-plane sapphire substrate. This layer was subsequently re-evaporated at a higher temperature to form mono- or few-layer MoS_2_ flakes. As a result, the close proximity re-evaporation enabled the growth of pristine MoS_2_ nanoflakes. Atomic force microscopy analysis confirmed the synthesis of nanoclusters/nanoflakes with lateral dimensions of over 10 μm and a flake height of approximately 1.3 nm, demonstrating bi-layer MoS_2_, whereas transmission electron microscopy analysis revealed triangular MoS_2_ nanoflakes, with a diffraction pattern proving the presence of single crystalline hexagonal MoS_2_. Raman data revealed the typical modes of high-quality MoS_2_ nanoflakes. Finally, we presented the photocurrent dependence of a MoS_2_-based photoresist under illumination with light-emitting diode of 405 nm wavelength. The measured current–voltage dependence across various luminous flux outlined the sensitivity of MoS_2_ to polarized light and thus opens further opportunities for applications in high-performance photodetectors with polarization sensitivity.

## 1. Introduction

Molybdenum disulfide (MoS_2_) belongs to the transition metal dichalcogenide (TMDC) family of two-dimensional (2D) layered materials characterized by strong covalent Mo–S bonds within each monolayer, stacked with weak Van der Waals forces between the neighboring monolayers. Unlike graphene, MoS_2_ is a semiconductor with a non-zero energy gap. This property allows MoS_2_ to complement graphene in various applications that require transparent semiconductor behavior, such as optoelectronics and energy harvesting [1,2,3,4,5,6]. Therefore, to date, MoS_2_ is the most intensely studied 2D material beyond graphene, which has already been employed in the fabrication of sensitive photodetectors, light-emitting diodes, field effect transistors, and many others [7,8,9,10]. When the dimension of MoS_2_ changes from a three-dimensional (bulk) into a two-dimensional form, the band structure transforms from an indirect to direct bandgap semiconductor and as a result, MoS_2_ demonstrates exceptional properties, like high carrier mobility and excellent optical transparency, an excellent combination for developing ultra-broadband photodetectors [11,12,13]. Moreover, MoS_2_ shows beneficial electronic and quantum characteristics which permits the downscaling of MoS_2_-based optoelectronic devices, logical elements, flexible electronics, and photodetectors [8]. As the most technologically mature TMDC, the photodetection capabilities of MoS_2_ have been proven in a variety of architectures including photodiodes and phototransistors; for example, MoS_2_ atomic layers can be used as a channel or a gate dielectric for making atomically thin field-effect transistors (FETs) [14]. Due to its unique properties, there are many research areas for 2D MoS_2_, which has great potential for future nanoscale optoelectronic devices.

Over the years, considerable effort has been made to synthesize MoS_2_ films with a controllable number of layers and on a large scale [5,14,15,16]. Mechanical exfoliation has been proven as the most widely used technique; however, it is not appropriate for large-scale production due to the limited monolayer coverage and difficult control of the number of layers [17,18]. As an alternative, bottom-up approaches, such as chemical vapor deposition, have been utilized to produce large-scale MoS_2_ films [19,20,21,22]. On the other hand, the thermal evaporation method offers several advantages such as the production of highly uniform thin films over large areas and reliable control over the deposition rate and thickness of the films [23,24,25]. Another method involves direct sulfurization of pre-deposited Mo layer in order to achieve a thin film surface [26]. Seeding promoter-controlled growth of molybdenum disulfide has also researched [27]. These methods are capable of producing very good quality MoS_2_ layers; however, achieving large-area MoS_2_ thin films is challenging.

To address these challenges, here, we present a novel and efficient synthesis method for MoS_2_ nanoflakes through close proximity re-evaporation of bulk MoS_2_ layers. Unlike other methods that require hydrogen (H_2_) or hydrogen sulfide (H_2_S) gases, our technique uses only argon (Ar) as a carrier gas, which eliminates the need for reactive and hazardous gases, significantly enhancing the safety and simplicity of the process. Additionally, it does not require any additional precursors, streamlining the synthesis procedure and reducing material costs [28,29,30]. This approach also ensures a more controlled and uniform deposition of MoS_2_ flakes, leading to higher quality and consistency in the resulting films. Additionally, the re-evaporation process allows for better control over the thickness and layer number, facilitating the production of ultra-thin and highly uniform films over a large area. This method is cost-effective and scalable, making it suitable for industrial applications where large-scale, high-quality MoS_2_ films are required. Furthermore, the elimination of toxic gases and the use of a simpler set-up can reduce production costs and environmental impact, making this method a more sustainable and economically viable option for the fabrication of next-generation optoelectronic devices.

## 2. Materials and Methods

The synthesis of MoS_2_ flakes was achieved through a precisely controlled re-deposition process using a low-pressure chemical vapor deposition technique (LPCVD). The method involved two primary steps. Firstly, a bulk MoS_2_ layer was initially synthesized on a c-plane sapphire substrate. Secondly, heating to higher temperature was applied to induce re-evaporation and cause the re-deposition of a monolayer MoS_2_ film.

**Figure 1:**
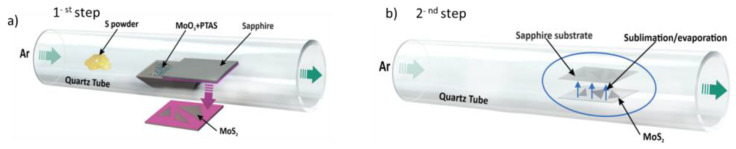
(**a**) Initial synthesis of a bulk MoS_2_ layer on a c-plane sapphire substrate; (**b**) the subsequent heating to achieve re-evaporation and re-deposition of monolayer MoS_2_ film.

Initially, a bulk multilayer MoS_2_ was synthesized and placed within the first zone of the LPCVD furnace, as depicted in Figure 1a. Directly above the bulk MoS_2_ layer, a pristine c-plane sapphire substrate was positioned on a clean boat to ensure that the re-deposited MoS_2_ layer would be formed on a well-defined surface.

The temperature of the first zone was precisely increased to 1000 °C and maintained for two hours, a critical period for the initial decomposition and re-evaporation of the bulk MoS_2_ layer. During the high-temperature phase, a constant argon gas flow rate of 25 standard cubic centimeters per minute (sccm) was maintained. The controlled flow of argon fulfilled a dual function in the experimental set-up. Firstly, it facilitated the transport of evaporated MoS_2_ seeds from the bulk material to the sapphire substrate, enabling the seeds to re-deposit effectively. Secondly, the regulated argon flow constrained the amount of seeding material, ensuring that a limited number of layered flakes could form on the substrate. This precise control was crucial for achieving the desired deposition and formation of MoS_2_ flakes on the sapphire surface.

As a result of the elevated temperature, the bulk MoS_2_ layer underwent sublimation/evaporation, causing the MoS_2_ flakes to re-deposit onto the blank c-plane sapphire substrate, as shown in Figure 1b. This process facilitated the formation of a pristine MoS_2_ monolayer.

The surface topology and thickness of deposited layers were examined by atomic force microscopy (AFM) using MFP-3D (Asylum Research, Oxford Instruments, Santa Barbara, CA 93117, USA).

The morphology and qualitative elemental analysis of the MoS_2_ films were studied with a field emission scanning electron microscope (JEOL IT800SHL, JEOL Ltd., Tokyo, Japan) through both secondary and backscattered electron detectors placed within the in-chamber and in-lens microscope columns. The topography of the MoS_2_ films was clearly revealed by the 5-segment versatile backscattered electron detector.

Observation of the morphology, microstructure, and phase composition on a nanoscale was allowed by a transmission electron microscope (JEOL JEM 2100) (JEOL Ltd., Tokyo, Japan) at 200 kV accelerating voltage. Three modes of the microscope were applied—Bright Field Transmission Electron Microscopy (BF-TEM), Selected Area Electron Diffraction (SAED), and High-Resolution TEM (HR TEM).

Raman analysis was performed in backscattering geometry at a HORIBA Jobin Yvon Labram HR visible spectrometer (HORIBA Ltd., Kyoto, Japan) visible spectrometer equipped with a Peltier-cooled CCD detector. The He–Ne laser (emitting at 633 nm) was focused on a spot of about 1 µm in diameter on the sample surface using microscope optics with an objective of 100× magnification. A Si standard was used to calibrate the frequency and the Raman line parameters were determined by means of fitting to Voigt profiles.

The optical transmittance spectra in the wavelength range of 200 nm to 800 nm were measured at room temperature using an Ultraviolet–Visible–Near-infrared (UV-VIS-NIR) spectrophotometer (Cary 5E (Varian, Sydney, Australia)). The photoluminescence (PL) spectrum of MoS_2_/sapphire was measured using the Spectrofluorometer FluoroLog3-22, Spectrofluorometer FluoroLog3-22, Horiba JobinYvon (HORIBA Jobin Yvon S.A.S., 91165 Longjumeau cedex, France) with the excitation wavelength at 445 nm.

To measure the photosensitivity, silver electrodes were deposited on the top of the MoS_2_ flakes (island structure), using a 25 µm mesh mask. Such a MoS_2_-based configuration was irradiated with a 405 nm light-emitting diode (LED) to study the photosensitivity under linearly polarized light. The assembled structure allowed for the measurement of the polarization sensitivity, as the light’s polarization angle could vary relative to the orientation of the electrodes. Measurements were conducted using the Keithley 230 Programmable Voltage Source and 617 Keithley meter (Programmable Electrometer (Tektronix, Inc. Beaverton, OR, USA)).

## 3. Results and Discussions

### 3.1. Optical, SEM and AFM Images and Surface Morphology

**Figure 2:**
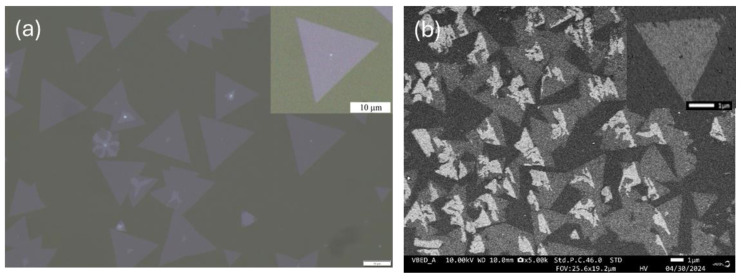
(**a**) Optical microscope image of MoS_2_ flakes on sapphire (×100), scale bar 10 µm and (**b**) SEM image in backscattered electrons.

The MoS_2_ flakes were equilateral-triangle-like in shape with almost uniform side lengths of around 8–10 µm. Careful analysis of a large set of optical and electron microscope images revealed well-shaped MoS_2_ triangles, with distinct sharp edges. Figure 2a shows the optical microscope images of the MoS_2_ film with triangular shape flakes distributed along the substrate. The SEM image in backscattered electrons is presented in Figure 2b.

**Figure 3:**
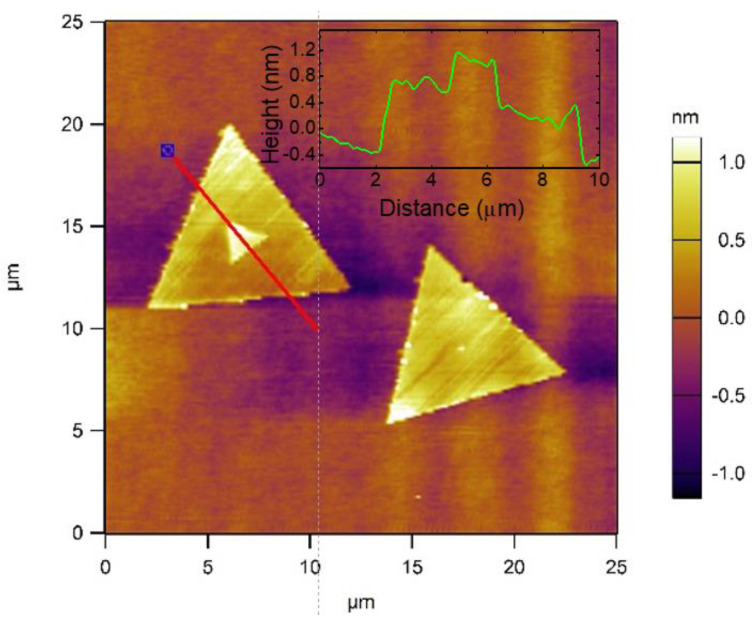
AFM image of MoS_2_ flakes on sapphire with the height profile (inset graph).

AFM measurements were taken on MoS_2_ flakes at random locations marked by a red line as shown in Figure 3. The height profile, with a thickness of around 1.3 nm, is presented as an inset in Figure 3, demonstrating a bilayer MoS_2_ flake (a single layer of MoS_2_ is reported to be approximately 0.65 nm thick) [11,31,32].

### 3.2. Transmission Electron Microscopy (TEM) Analysis

**Figure 4:**
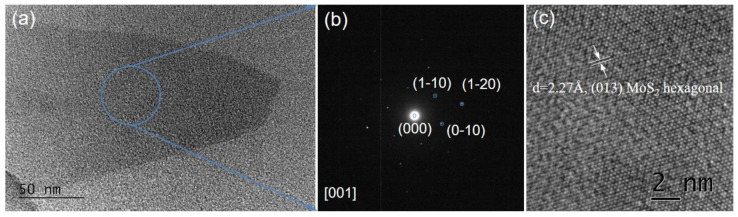
(**a**) Bright Field TEM micrograph of MoS_2_ flake at magnification 100,000×; (**b**) The corresponding Selected Area Electron Diffraction pattern of the central area of the MoS_2_ flake, presented in (**a**); (**c**) High-Resolution TEM image of the MoS_2_ flake, presented in (**a**).

The morphology visualization, microstructure, and phase composition analysis of the MoS_2_ flakes were registered and analyzed using TEM and are presented in Figure 4. At the nanoscale level, the triangular shape of the flakes, as determined by optical, scanning electron, and atomic force microscopy (see Figure 2 and Figure 3, respectively), appeared deformed (Figure 4a), which most likely occurred in the process of layer transfer from the deposition substrate to the copper TEM grid, serving for introducing the studied material in the apparatus chamber. The diffraction pattern (Figure 4b), collected from the central area of the flake and as presented in Figure 4a, allowed us to determine the phase composition of the sample as hexagonal MoS_2_ with P 63/mmc Space group and cell parameters a = 3.15000 Å and c = 12.30000 Å, according to Crystallography Open Database Entry #96-101-0994. The SAED pattern consisted of one set of diffraction spots, such that the closest of them to the central beam were arranged in a hexagonal configuration, thus demonstrating that the flake was composed of a single layer of MoS_2_. The indexing of the electron diffractogram revealed the orientation of the flake to be along the zone axis [001], which is characteristic for growth of MoS_2_ on a c-plane oriented sapphire substrate. The high-resolution TEM image (Figure 4c) revealed the (013) family of MoS_2_ crystal planes with their characteristic interplanar spacing of 2.27 Å and confirmed the phase composition as hexagonal MoS_2_.

### 3.3. Raman Analysis

**Figure 5:**
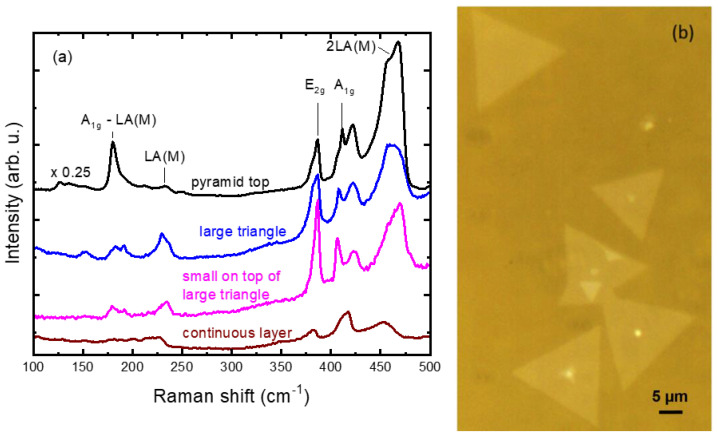
(**a**) Raman spectra of various MoS_2_ flakes (indicated in the plot) on sapphire. (**b**) Optical micrograph depicting the objects from which the two middle spectra were recorded.

Raman spectra of the MoS_2_ nanoflakes are shown in Figure 5a with two characteristic Raman peaks—one around 387 cm^−1^ corresponding to the in-plane E_2g_ mode and one around 407 cm^−1^ corresponding to the out-of-plane A_1g_ mode of MoS_2_. As the exciting laser energy nearly coincided with the direct band gap (∼1.96 eV) at the K point, the Raman spectra also contained resonantly enhanced combination and forbidden modes as well as weak satellites at the low-frequency side of the E_2g_ and the A_1g_ line [33]. The intensive asymmetric band at ≈460 cm^−1^ was assigned to a second-order zone-edge vibration 2LA(M) with a possible contribution of the Raman forbidden phonon A_2u_ at its high-frequency side [34]. The band centered at 180 cm^−1^ was assigned to an A_1g_ (M) − LA(M) difference process, and the peak at 415–420 cm^−1^ had a complex origin assumed to involve the Raman–inactive E_1u_ phonon [35].

The uppermost spectrum in Figure 5a was taken from the top of a pyramid-like flake, the two middle spectra stem from triangular-like flakes (depicted in Figure 5b) and the bottom spectrum was taken from a continuous layer. The frequency distance between the vibrational modes E_2g_ and A_1g_ allowed us to evaluate the number of MoS_2_ monolayers, as extensively reported in the literature [36,37,38]. In our case, the measured E_2g_−A_1g_ frequency distance Δω amounted to ≈20 cm^−1^ for the triangular-like flakes which, according to the literature [39], proved the growth of monolayer MoS_2_. The full width at half maximum (FWHM) of the E_2g_ peak may be used as an indicator for crystalline quality. The two Raman active modes formed relatively sharp lines with FWHM of the E_2g_ and A_1g_ peaks of about 6 and 5 cm^−1^, respectively. This suggested a good crystalline quality in the synthesized MoS_2_ [16]. In the uppermost spectrum, Δω = 25 cm^−1^, which was consistent with the increased number of MoS_2_ monolayers beneath the pyramid top, while the peaks in the continuous-layer spectrum were largely smeared out thus hindering the frequency determination.

### 3.4. Optical Absorbance and Photoluminescence

**Figure 6:**
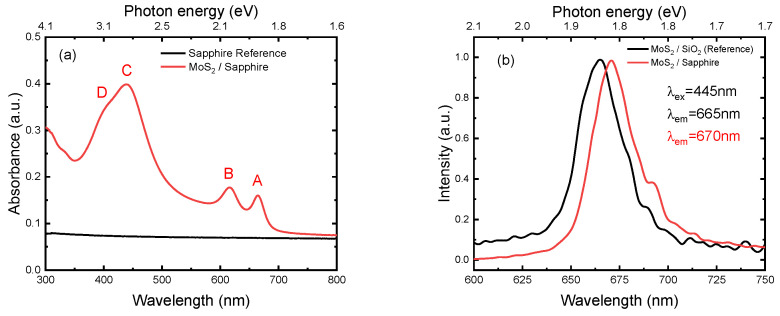
(**a**) Optical absorbance spectra of MoS_2_ layer on sapphire (sapphire substrate also measured for reference) and (**b**) photoluminescence of MoS_2_ layer on sapphire compared with MoS_2_ layer on SiO_2_ (commercially available reference).

Ultraviolet–visible spectroscopy was used to characterize the MoS_2_ layer and the optical absorbance of the MoS_2_ monolayer flake in air. To calculate the absorbance from transmittance, the Beer Lambert Law for transmittance equation was used as displayed in Figure 6a. Four prominent absorption peaks were observed in the MoS_2_ monolayer, labelled as (A), (B), (C), and (D) in Figure 6a, and they were located at 664 nm (1.87 eV), 616 nm (2.01 eV),438 nm (2.83 eV), and 405 nm (3.06 eV), respectively. The absorbance spectra had two prominent narrow peaks occurring at wavelengths ~616 nm and ~664 nm that corresponded to the absorption due to the direct transitions at the K point of the Brillouin zone, associated to the generation of the B and A excitons, respectively [40,41,42]. The strong spin–orbit coupling in MoS_2_ had split the valence band into two sub-bands at the Κ point in the Brillouin zone within the transition metal sulfides, which made the two exciton states of the inter-band transition, peak A and peak B. The two peaks (denoted as A and B) corresponded with the excitons formed due to the interaction between an excited electron in the lowest conduction band and an excited hole in the spin–orbit (SO) split valence band at the K point. The spectra also showed a broad peak around 438 nm. Peak C resulted from the contribution of chalcogen orbitals to transitions far away from the Κ point, direct Mo(d)↔S(p) excitations [43]. The peak at 3.06 eV, denoted as D, corresponded to transitions between Van Hove singularities at the M point, which exhibited a very intensive S(p) character in the valence band and a very intensive Mo(d) character in the conduction band. Recent reflectance and photocurrent spectroscopy experiments, however, presented this feature (referred to as C-exciton peak) whose origin remains a subject of debate [44].

Photoluminescence spectroscopy is a direct method for measuring the band gap, as the energy absorbed by the MoS_2_ layer results in the emission of light. PL spectroscopy measurement (Figure 6b) was performed using an excitation wavelength of 445 nm, and emission peaks were detected at 670 nm and 665 nm for MoS_2_/sapphire and MoS_2_/SiO_2_, respectively, in good agreement with [45,46]. The strong photoluminescence emissions unambiguously indicated the presence of monolayer MoS_2_ [47]. Xiao Li et al. reported that the PL spectra were intrinsically related to the number of layers and the thickness of the nanosheets of MoS_2_ [4]. Zhen Li also confirmed that the photoluminescence of monolayer MoS_2_ was significantly more intense than that of the bulk layer, and the introduction of layers into the monolayer decreased the intensity considerably [48].

### 3.5. MoS_2_-Based Photoresistor

The interest in 2D TMDCs in general and MoS_2_ in particular is that a single layer of MoS_2_ atoms is a direct band gap semiconductor whereas the bulk form is an indirect band gap semiconductor. Thus, single-layer MoS_2_ emits light when illuminated with energy above the band gap. This property holds the promise of being able to fabricate electrooptic devices from single-layer MoS_2_ or other TMDs and has therefore been the subject of intense research and development.

**Figure 7:**
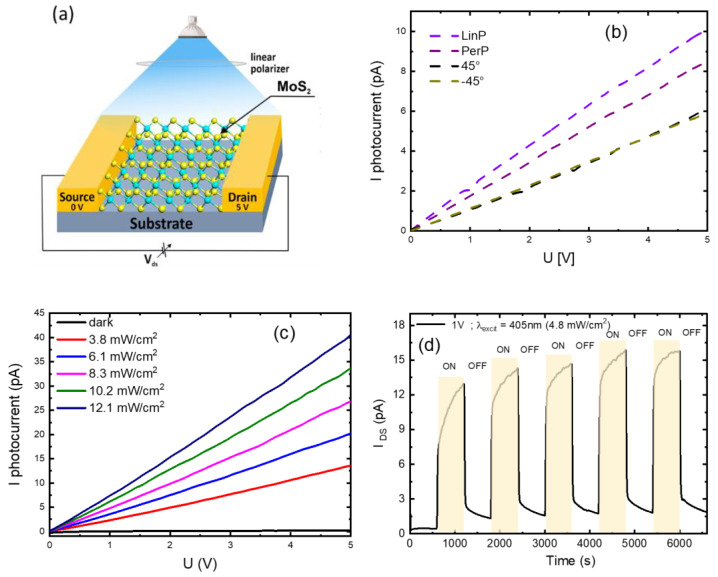
(**a**) Schematic diagram of MoS_2_ photoresist; (**b**) I_DS_-V_DS_ characteristic of MoS_2_ as a function of light polarization; (**c**) I_DS_-V_DS_ characteristic of MoS_2_ as a function of light intensity of non-polarized light (**d**) “On” and “off” cycles of I_DS_ at fixed voltage of 1 V.

To study the photoresponse, a MoS_2_-based phototransistor configuration was assembled (Figure 7a). Linearly polarized light with a wavelength of 405 nm was used to irradiate the sample, with a fixed intensity of 2.4 mW/cm^2^. Photocurrent–voltage (I_DS_–V_DS_) measurements were performed using an LED operating at a wavelength of 405 nm and a power density of 4.8 mW/cm^2^.

Linear I_DS_–V_DS_ dependence and significant photoresponse under 405 nm light irradiation were observed. Figure 7b shows the dependence of polarization sensitivity of the linearly polarized light, measured relative to the orientation of the electrodes. The dashed lines indicate the polarization sensitivity to the linearly polarized light. “LinP” corresponds to light polarization parallel to the electrodes, while “PerP” indicates light polarization perpendicular to the electrodes. The terms 45° and −45° denote distinct angles of linear polarization, revealing the specific alignment of the MoS_2_ layer during the growth process. It is evident that the photocurrent varies with the polarization angle, demonstrating the anisotropic nature of the MoS_2_ behavior.

We assume the mechanism was related to the anisotropy of charge carrier’s generation depending on the light polarization. The photocurrent was at maximum when the polarization direction was perpendicular to the MoS_2_ channel direction, and the photocurrent was minimum when the two directions were parallel. This polarization dependence can be explained as being due to the anisotropic electron–photon interaction. Similar behavior of MoS_2_-based photodetectors has been reported in the literature [49,50]. Moreover, this anisotropic behavior is consistent with other studies on polarization-sensitive photodetectors, such as the GaTe/MoS_2_ Van der Waals heterojunctions, which exhibited fourfold anisotropy with a high polarization ratio due to their highly anisotropic monoclinic structure [51].

Figure 7c illustrates the I_DS_–V_DS_ dependencies across various luminous flux power levels. As the power density increased from 3.8 mW/cm^2^ to 12.1 mW/cm^2^, the photocurrent also increased, indicating a direct relationship between the incident light power and the generated photocurrent. This relationship underscores the material’s sensitivity and responsiveness to different light intensities. Similar observations have been reported in WSe_2_-based devices, where interlayer coupling induces distinct linear dichroism [52].

In addition, at a fixed voltage of 1 V, the response of light irradiation as a function of time was measured (Figure 7d). The observed increase in the signal over the time can be attributed to the charging of capacitance distributed across a substantial resistance. This behavior aligns with the results presented in [10,53], where a similar increase in microcurrent was observed, and an equivalent circuit model was proposed, characterized by the relationship C=R=C, emphasizing the interplay between the capacitance and resistance.

The calculated values of performance indicators in terms of responsivity, external quantum efficiency (EQE), and detectivity at voltage of 5 V were, respectively, 16.47 mA/W, 5.05%, and 3.35 × 10^8^ Jones, which are much smaller than those for the O–WS_2_/WS_2_ photodetectors studied by [49,54].

The charging of capacitances through a large resistance, causing an increase in current at fixed light intensity, can be utilized in optical switches, regulators, or light filters. The above characteristic makes the MoS_2_-based phototransistor a promising candidate for applications in optoelectronic devices where the precise control of light-induced current is crucial.

## 4. Conclusions

We have successfully demonstrated the synthesis of scalable monolayer and few-layer MoS_2_ films and flakes through an LPCVD process, utilizing the proximity evaporation of an MoS_2_ film precursor. This cost-effective and scalable method is suitable for industrial applications requiring large-scale, high-quality MoS_2_ films. Additionally, the elimination of toxic gases and the use of a simple set-up reduce production costs and environmental impact, making this approach sustainable for fabricating next-generation optoelectronic devices.

## Data Availability

Data are contained within the article.
